# Pulmonary Arterial Hypertension Requiring Medication Is Associated With Higher Prevalence of Thrombocytopenia in Pediatric Patients

**DOI:** 10.1002/ppul.71252

**Published:** 2025-08-19

**Authors:** Tanya Reyna, Catherine Agarwal, Abrar Mamun, Jessica Dae, Yusuf Ozcan, Michael J. Angtuaco, Taha Bat, Erhan Ararat

**Affiliations:** ^1^ Department of Internal Medicine, Division of Hematology/Oncology University of Texas Southwestern Medical Center Dallas Texas USA; ^2^ Biological Sciences University of Texas at Dallas Dallas Texas USA; ^3^ Department of Pediatrics University of Arkansas for Medical Sciences Dallas Texas USA

**Keywords:** bone marrow, hemoglobin, leukocytes, lungs, platelets

**Abbreviations**
PAH: pulmonary arterial hypertension

To the Editor,

Although platelet biogenesis is traditionally thought to occur in the bone marrow, recent studies elucidate the role of the lungs as a site for thrombopoiesis [1]. While platelet‐producing megakaryocytes in the pulmonary circulation are generated in the bone marrow and spleen, they can be trapped in the lung vasculature, where platelet release occurs (Figure 1). If the lung endothelium is injured, such as in pulmonary arterial hypertension (PAH), this process could be impeded. In fact, there is evidence that up to 20% of adults with idiopathic PAH (iPAH) experience thrombocytopenia [2], or a platelet count < 150,000/μL, which is associated with reduced 5‐year survival [2].

**FIGURE 1:**
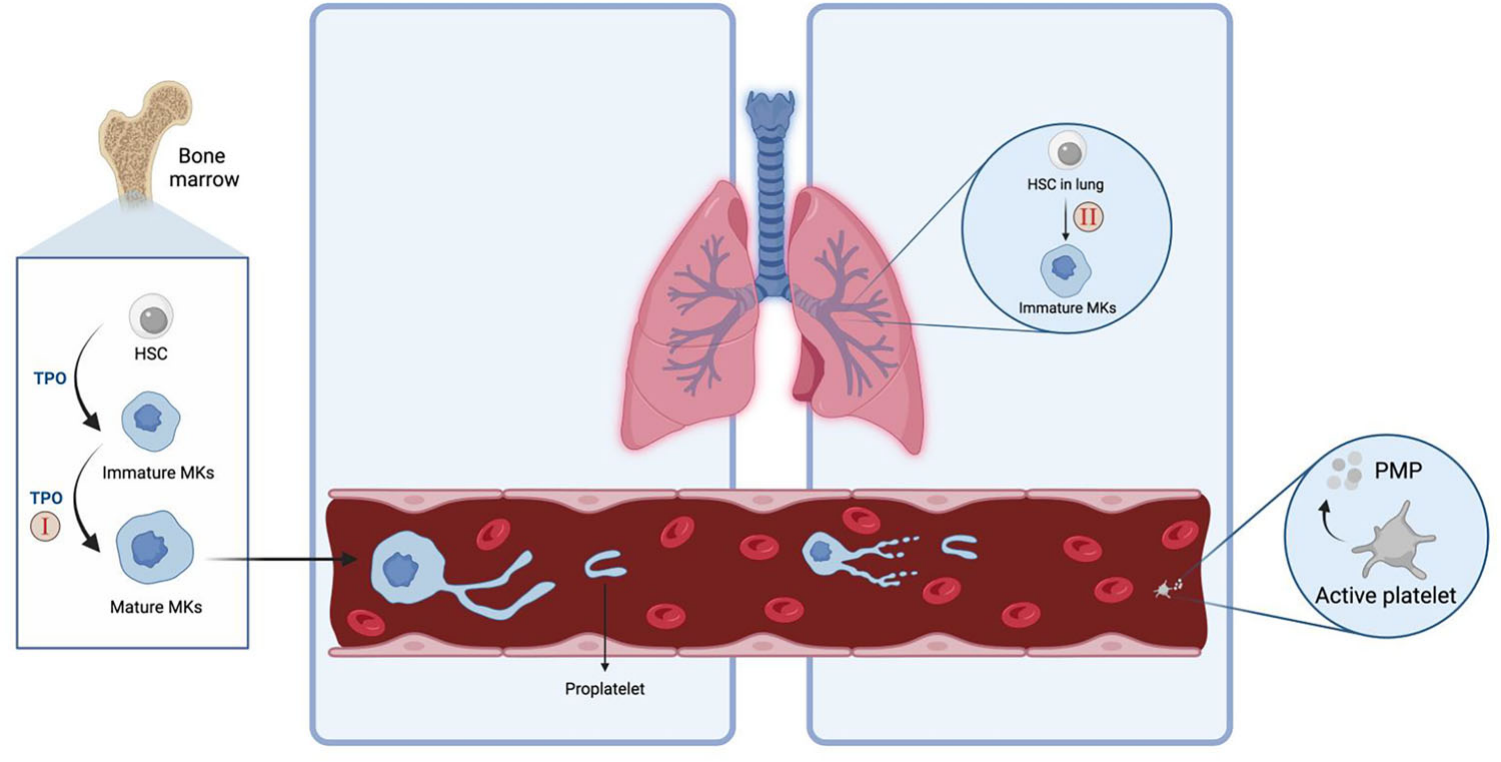
Proposed locations and mechanisms of thrombopoiesis in the human body. Mechanism I displays proplatelet development/release and megakaryocyte egress originating from the bone marrow. Mechanism II suggests that proplatelet shedding can also occur in the lungs. The latter mechanism may potentially be affected by PAH‐mediated endothelial dysfunction and signaling imbalances in the lung vasculature, resulting in abnormal platelet counts. [Color figure can be viewed at wileyonlinelibrary.com]

There is also evidence that up to 43% of pediatric patients with PAH demonstrate abnormal platelet aggregation [3]. Despite untreated PAH having a higher mortality rate in children than adults [4], the association between platelet count changes and PAH is not well understood in the pediatric population. Therefore, this study aims to compare the median platelet count among pediatric patients with varying degrees of PAH severity, categorized according to vasodilator therapies.

The data used in this study was collected on November 27, 2023, from the TriNetX Research Network, which provided access to electronic medical records (diagnoses, procedures, medications, laboratory values, genomic information) of approximately 140 million patients from 40 health care organizations. Since this study is a retrospective analysis of a deidentified database, it was exempt from review by the University of Arkansas Medical Sciences (UAMS) Institutional Review Board, a determination refreshed in December 2020.

We identified 1832 patients < 18 years old who were (1) diagnosed with PAH and (2) had a platelet count, leukocyte count and hemoglobin level within 3 months of their first PAH diagnosis. Patients were divided into two groups: (1) those who used no anti‐PAH medications and (2) those who used anti‐PAH therapy, including sildenafil with or without another anti‐PAH medication.

A 2:1 greedy propensity score match with a caliper width of 0.2 was performed on the two groups to elucidate the presence of and control for systematic differences in demographic characteristics and confounding comorbidities. Following propensity score matching, the no medications group (*n* = 872) and anti‐PAH medication group (*n* = 436) were compared based on platelet counts (primary outcome) followed by leukocyte counts and hemoglobin levels (secondary outcomes) using Wilcoxon rank sum tests, Pearson's Chi‐squared tests, and/or Fisher's exact tests.

The two groups had similar racial and gender demographic characteristics (Table S1). Approximately half of the participants were identified as white and a quarter as Black or African American. Half of the participants were identified as male. Before propensity score matching, the median age for both groups was 1 year old, though the age range was wider in the untreated group. A diagnosis of bronchopulmonary dysplasia (BPD) was found at similar rates in the treated group (*n* = 76, 17%) and the untreated group (*n* = 208, 15%). The treated group had a higher rate of congenital heart defect diagnoses (*n* = 322, 74%) compared to the untreated group (*n* = 763, 55%).

After matching on baseline characteristics to eliminate confounding from age and congenital heart disease status, the treated group was found to have a significantly lower platelet count compared to the untreated group (*p* < 0.001). The treated group also had a higher rate of diagnosed thrombocytopenia compared to the untreated group (*p* = 0.009), but the severity of thrombocytopenia was similar for both groups. Upon analysis of white blood cell and hemoglobin counts in the thrombocytopenic patients of each group, both groups had similar rates of leukopenia (13% of treated, 9.6% of untreated) and anemia (29% of treated, 26% of untreated).

Similar to previous observations in adults with PAH, there is a significant association between PAH and thrombocytopenia in pediatric patients. Patients on anti‐PAH medications had significantly lower platelet counts and were more likely to have thrombocytopenia compared to those not on anti‐PAH therapy (Table 1). Notably, this study also revealed that leukocyte counts and hemoglobin values were not significantly different between the thrombocytopenic patients of the two groups (Table 1). These key findings suggest that in PAH that is severe enough to be treated, the observed thrombocytopenia may be due to compromised platelet shedding in the lung vasculature rather bone marrow failure, as the latter would otherwise result in decreased counts of platelets, leukocytes, and hemoglobin (Figure 1, Mechanism II).

**TABLE 1:** Comparison of platelet counts and thrombocytopenia characteristics by PAH treatment status.

Characteristic	No Medications N = 872^a^	Anti‐PAH Medications N = 436^a^	*p* value^b^
Platelet count (10^3^/µL)	288 (202, 392)	251 (163, 363)	**< 0.001**
Thrombocytopenia	125 (14%)	87 (20%)	**0.009**
Thrombocytopenia Severity	0.2		
Mild (100‐150 ×103/µL)	79 (63.2%)	59 (67.8%)	
Moderate (100‐150 ×103/µL)	44 (35.2%)	24 (27.6%)	
Severe ( < 50 ×103/µL)	2 (1.6%)	4 (4.6%)	
Other Cell Counts in Thrombocytopenic Patients			
Leukocyte count (10^3^/µL)	10 (7, 15)	9 (7, 13)	0.6
Leukopenia	12 (9.6%)	11 (13%)	0.5
Hemoglobin (g/dL)	12.0 (10.5, 13.7)	11.8 (10.2, 13.5)	0.5
Anemia	32 (26%)	25 (29%)	0.6

^a^ Median (Q1, Q3); n (%).

^b^ Wilcoxon rank sum test; Pearson's Chi‐squared test; Fisher's exact test.

The literature suggests that a significant portion of platelet production occurs in lung vasculature [1]. Upon histological section, several studies have documented the presence of megakaryocytes in the pulmonary circulation that appear to be active in platelet production [5]. There is also a greater concentration of megakaryocytes found in pulmonary arterial blood when compared to aortic blood. Approximately 98% of megakaryocytes entering pulmonary circulation did not leave as megakaryocytes or fragments of megakaryocytes, suggesting that platelet production occurred within the lungs [6]. Estimates of the proportion of platelet production from the lungs ranges from 7% to 70% [1] and warrants further investigation.

The process of platelet shedding in lung vasculature is related to the direct contact of megakaryocytes with endothelial cells and a variety of signaling factors that are released into the microenvironment of the vasculature. Endothelial dysfunction and signaling imbalance in the lung vasculature are well documented in PAH and thus implicate a potential role in impaired platelet release in the lungs (Figure 1, Mechanism II). However, future studies are needed to investigate these potential specific signaling abnormalities and how they may contribute to reduced platelet shedding in PAH.

A major limitation of this study was the use of medication status to distinguish the severity of PAH between groups rather than direct measurements of PAH, such as echocardiography and pulmonary capillary wedge pressure. Future studies are needed to directly correlate thrombocytopenia with PAH severity, and to incorporate longitudinal data to better understand the relationship between PAH and platelet counts over time. More thorough consideration of confounding variables, such as disease severity, potential comorbidities not considered in the scope of this study, and medication adherence will also enhance the accuracy of further research. Notably, studies of adult patients have found thrombocytopenia to be an independent predictor of mortality in PAH [2]. This study's documentation of thrombocytopenia in the pediatric PAH population warrants further investigation into these aforementioned clinical implications of thrombocytopenia for these patients.

## Author Contributions

**Tanya Reyna:** writing – original draft, writing – review and editing, visualization, investigation. **Catherine Agarwal:** writing – review and editing, writing – original draft, visualization, investigation. **Abrar Mamun:** writing – original draft, writing – review and editing, investigation, visualization. **Jessica Dae:** writing – review and editing, writing – original draft, visualization, investigation. **Yusuf Ozcan:** writing – original draft, writing – review and editing. **Michael J Angtuaco:** conceptualization, investigation, methodology, resources, supervision. **Taha Bat:** funding acquisition, investigation, conceptualization, methodology, project administration, resources, supervision. **Erhan Ararat:** software, formal analysis, visualization, validation, methodology, conceptualization, investigation, resources, supervision, data curation.

## Conflicts of Interest

The authors declare no conflicts of interest. Preliminary study data were previously presented in the form of a poster, with an associated abstract, at the American Society of Hematology Annual Meeting (2024). It was also presented as a poster at the American Medical Association Interim Meeting Poster Showcase (2024).

## Supporting information


**Supplemental Table 1.** Baseline characteristics of study population before and after propensity score matching.

## Data Availability

The data that support the findings of this study are available from TriNetX LLC. Restrictions apply to the availability of these data, which were used under license for this study. Data are available from the author(s) with the permission of TriNetX LLC.
