# Supplementary material for: Pulmonary Arterial Hypertension Requiring Medication Is Associated With Higher Prevalence of Thrombocytopenia in Pediatric Patients
Source: Pediatr Pulmonol. 2025 Aug 19;60(8):e71252. doi: 10.1002/ppul.71252 (PMC12363154; doi:10.1002/ppul.71252)
Supplement: Supplementary file 1 — Supplemental Table 1. Baseline characteristics of study population before and after propensity score matching. [file PPUL-60-0-s001.docx]

Supplemental Table 1. Baseline characteristics of study population before and after propensity score matching

|  | **Before Match** | | | **After Match** | | |
| --- | --- | --- | --- | --- | --- | --- |
| **Characteristic** | **No Medications** N = 1,396^1^ | **Anti-PAH Medications** N = 436^1^ | **p-value**^2^ | **No Medications** N = 872^1^ | **Anti-PAH Medications**  N = 436^1^ | **p-value**^3^ |
| Age | 1.0 (0.0, 5.0) | 1.0 (0.0, 2.0) | **<0.001** | 1.0 (0.0, 2.0) | 1.0 (0.0, 2.0) | 0.5 |
| Sex (Males) | 736 (53%) | 213 (49%) | 0.2 | 425 (49%) | 213 (49%) | >0.9 |
| Race |  |  |  |  |  | >0.9 |
| White | 743 (53%) | 223 (51%) |  | 443 (51%) | 223 (51%) |  |
| Black or African American | 315 (23%) | 111 (25%) |  | 203 (23%) | 111 (25%) |  |
| Unknown | 151 (11%) | 49 (11%) |  | 102 (12%) | 49 (11%) |  |
| Other Race | 116 (8.3%) | 36 (8.3%) |  | 86 (9.9%) | 36 (8.3%) |  |
| Asian | 49 (3.5%) | 13 (3.0%) |  | 30 (3.4%) | 13 (3.0%) |  |
| American Indian or Alaska Native | 13 (0.9%) | 4 (0.9%) |  | 8 (0.9%) | 4 (0.9%) |  |
| Native Hawaiian or Other Pacific Islander | 9 (0.6%) | 0 (0%) |  | 0 (0%) | 0 (0%) |  |
| Prematurity/BPD | 208 (15%) | 76 (17%) | 0.2 | 143 (16%) | 76 (17%) | 0.6 |
| Congenital Heart Disease | 763 (55%) | 322 (74%) | **<0.001** | 634 (73%) | 322 (74%) | 0.7 |
| ^1^Median (Q1, Q3); n (%) | | | | | | |
| ^2^Wilcoxon rank sum test; Pearson's Chi-squared test | | | | | | |
| ^3^Wilcoxon rank sum test; Pearson's Chi-squared test; Fisher's exact test | | | | | | |
